# 

**Published:** 1829-07

**Authors:** 


### THE LONDON MEDICAL AND PHYSICAL JOURNAL.

S.G.O. INDEXED

EDITED BY

JOHN NORTH, ESQ. F.L.S.

MEMBER OF THE ROYAL COLLEGE OF SURGEONS,
AND OF THE MEDICAL AND CHIRURGICAL SOCIETY OF LONDON;

AND

JOHN WHATLEY, M.D. A.M.

MEMBER OF THE ARCADIA OF ROME;
MEMBER EXTRAORDINARY OF THE ROYAL MED. SOCIETY OF EDINBURGH;
MEMBER OF THE MEDICAL AND CHIRURGICAL SOCIETY OF LONDON;
AND OF THE MEDICAL SOCIETY OF LONDON.

(VOL. LXII.)

NEW SERIES, VOL. VII.

Et quoniam variant morbi, variabimus artes;

Mille mali species, mille salutis erunt.

Surgeon Genl's Office LIBRARY 45595

LONDON:

JOHN SOUTER, 73, ST. PAUL'S CHURCH-YARD,

AND TO BE HAD OF ALL THE MEDICAL BOOKSELLERS.

1829.

W1
LO 31

J. AND C. ADLARD, PRINTERS,
BARTHOLOMEW CLOSE.

### MONTHLY LIST OF MEDICAL BOOKS.

[*Medical Works cannot be entered on this List except a copy be sent for the purpose; the titles of Books having frequently been transmitted to us, as published, which have not appeared for weeks, or even months, after.*]

The Influence of Climate in the Prevention and Cure of Chronic Diseases, more particularly of the Chest and Digestive Organs; comprising an Account of the principal Places resorted to by Invalids in England and the South of Europe; a comparative Estimate of their respective Merits in particular Diseases; and general Directions for Invalids while travelling or residing Abroad. By James Clark, m.d. &c.—8vo. pp. 328. Underwood.

A Treatise on Syphilis, in which the History, Symptoms, and Method of Treating every form of that Disease are fully considered. By JOHN BACOT, Surgeon to the St. George's and St. James's Dispensary, and lately Surgeon to the Grenadier Regiment of Foot Guards.—8vo. pp. 280. Longman and Co. London, 1829.

An Account of some of the most important Diseases peculiar to Women. By ROBERT GOOCH, M.D.—8vo. pp. 432. Murray, London.

Answer to "Observations on the Phrenological Development of Burke, Hare, and other atrocious Murderers, &c. by Thomas Stone, Esq. &c." By GEORGE COMBE.—Anderson, Edinburgh; and Simpkin and Marshall, London, 1829.

A Rejoinder to the Answer of G. Combe, Esq. to "Observations on the Phrenological Development of Burke, &c." By THOMAS STONE, Esq. President of the Royal Medical Society of Edinburgh.—1829.

Hints for the Examination of Medical Witnesses. By JOHN GORDON SMITH, M.D. M.R.S.L. Professor of Medical Jurisprudence in the University of London.—12mo. pp. 138. Longman and Co.

### NOTICES.

*Communications have been received from* Mr. WALLER, Mr. LEONARD, Mr. CHENEVIX, Mr. HEMING, Mr. HIGGINBOTTOM, &c.

